# Supplementary material for: Chemical Profile and Multicomponent Quantitative Analysis for the Quality Evaluation of Toad Venom from Different Origins
Source: Molecules. 2019 Oct 6;24(19):3595. doi: 10.3390/molecules24193595 (PMC6804263; doi:10.3390/molecules24193595)
Supplement: Supplementary file 1 [file molecules-24-03595-s001.pdf]

Table 1. HPLC-ESI-Q-TOF-MS/MS data of the 157 compounds detected in methanol extracts of the Chansu.

No.	Compound class	TR/min	Ion form	Molecular formula	Detected	Expected	Error (ppm)	MS/MS fragments	Identification	Ref.
1	Amino acids	3.7	$[M+H]^+$	$C_6H_{15}O_2N_4$	175.1183	175.119	-3.7	175.1185, 158.0908, 130.0977, 116.0705, **70.0674**;	L-arginine	[1]
2	Amino acids	4.313	$[M+H]^+$	$C_{10}H_{19}O_5N_4$	275.134	275.135	-3.6	275.1346, 240.0975, 175.1186, 152.0702, **70.0676**;	Succinyl arginine	[1,2]
3	Amino acids	4.363	$[M+H]^+$	$C_{10}H_{17}O_4N_4$	257.1235	257.1244	-3.6	257.1240, 198.0753, **152.0703**, 71.0514;	Unknown	-
4	Amino acids	5.2	$[M+H]^+$	$C_{10}H_{17}O_4N_4$	257.1235	257.1244	-3.6	257.1231, 198.0751, **152.0701**, 71.0512;	Unknown	-
5	Vitamine	5.286	$[M+H]^+$	$C_6H_6O_2N$	124.039	124.0393	-2.5	124.0387, 106.0295, 80.0508, **78.0352**, 53.0413, 52.0234, 51.0327;	Nicotinic acid	[3]
6	Amino acids	5.466	$[M+H]^+$	$C_6H_{14}O_2N$	132.1014	132.1019	-3.8	**86.0997**, 69.0703, 58.0342, 56.0535;	Leucine	[3]
7	Amino acids	5.699	$[M+H]^+$	$C_{12}H_{23}O_5N_4$	303.1653	303.1663	-3.3	303.1651, 250.1173, 175.1177, 158.0911, 140.0806, 116.0699, **70.0670**;	Adipyl arginine isomer	[4]
8	Purine	6.022	$[M+H]^+$	$C_5H_5ON_4$	137.0452	137.0458	-4.3	137.0454, 119.0351, **110.0352**, 94.0407,	Hypoxanthine	[3]

								82.0411, 67.0312, 55.0329, 53.9990;		
9	Purine	6.556	$[M+H]^+$	$C_5H_5O_2N_4$	153.04	153.0407	-4.6	153.0382, **136.0130**, 110.0343, 81.0072, 55.0319, 53.0550;	Xanthine	[3]
10	Amino acids	7.103	$[M+H]^+$	$C_{11}H_{21}O_5N_4$	289.1498	289.1506	-2.9	289.1494, 240.0974, 197.0915, 175.1177, **115.0385**, 70.0671;	Glutaryl arginine	[4]
11	Amino acids	7.556	$[M+H]^+$	$C_{12}H_{23}O_5N_4$	303.1655	303.1663	-2.6	303.1662, 250.1183, 175.1188, 158.0924, 116.0713, **70.0681**;	Adipyl arginine	[1,4]
12	Hormone	7.697	$[M+H]^+$	$C_{10}H_{10}O_2N$	176.0698	176.0706	-4.6	176.0700, 161.0452, **133.0505**, 130.0644, 103.0545, 77.0403;	Indole-3-acetic acid	[3]
13[a]	Alkaloids	11.028	$[M+H]^+$	$C_{10}H_{13}ON_2$	177.1011	177.1021	-3.5	**160.0757**, 132.0809, 117.0575, 115.0546;	Serotonin	[1]
14	Alkaloids	11.162	$[M+H]^+$	$C_{11}H_{15}ON_2$	191.117	191.1179	-4.7	**160.0749**, 148.0741, 130.0642, 115.0544;	N’-methyl-serotonin	[1]
15	Amino acids	11.743	$[M+H]^+$	$C_{13}H_{25}O_5N_4$	317.1808	317.1819	-3.6	317.1812, 300.1546, 282.1441, 264.1337, 258.1329, 236.1387, **175.1186**;	Pimeloyl-arginine	[2,4]
16	Alkaloids	11.883	$[M+H]^+$	$C_{13}H_{19}ON_2$	219.1484	219.1492	-3.6	**160.0748,** 159.0674, 132.0805, 115.0544;	Bufotenidine	[1,5]

17	Nucleoside	12.206	$[M+H]^+$	$C_{10}H_{14}O_4N_5$	268.1029	268.104	-4.2	**136.0615**;	Adenosine	[3]
18	Alkaloids	12.892	$[M+H]^+$	$C_{12}H_{17}ON_2$	205.1327	205.1335	-4.1	**160.0754**, 159.0669, 132.0807, 117.0574, 115.0544, 58.0685	Bufotenine	[1,5]
19	Alkaloids	16.715	$[M+H]^+$	$C_{12}H_{15}ON_2$	203.1173	203.1179	-2.9	203.1175, **188.0938**, 173.0706, 155.0599, 146.0596;	Dehydrobufotenine	[1,5]
20	Alkaloids	16.75	$[M+H]^+$	$C_{12}H_{15}O_4N_2S$	283.0738	283.0747	-3.2	203.1170, **188.0934**;	Bufothionine	[1,5]
21	Amino acids	17.001	$[M+H]^+$	$C_{15}H_{29}O_5N_4$	345.212	345.2132	-3.6	345.2116, 275.1586, **258.1324**, 239.1379, 172.1072, 70.0676;	Azelayl arginine isomer	[4]
22	Amino acids	17.504	$[M+H]^+$	$C_{14}H_{27}O_5N_4$	331.1965	331.1976	-3.3	331.1956, 296.1593, 278.1485, 272.1481, 250.1532, 175.1179, **158.0917**, 112.0870, 70.0674;	Suberoyl-arginine	[1,4]
23	Amino acids	17.813	$[M+H]^+$	$C_{15}H_{29}O_5N_4$	345.212	345.2132	-3.6	345.2127, 303.1905, 189.1337, 172.1073, **129.1019**, 84.0820;	Azelayl arginine isomer	[4]
24	Amino acids	18.206	$[M+H]^+$	$C_{16}H_{31}O_5N_4$	359.2277	359.2289	-3.3	359.2273, 289.1751, **272.1484**, 253.1541, 203.1499, 172.1075, 115.0871, 70.0682;	Sebacyl arginine isomer	[4]
25	Amino acids	18.489	$[M+H]^+$	$C_{15}H_{29}O_5N_4$	345.212	345.2132	-3.6	345.2119, 292.1642, 264.1696, 175.1179,	Azelayl arginine isomer	[4]

								**158.0924**, 125.0957, 70.0676;		
26	Amino acids	19.314	$[M+H]^+$	$C_{15}H_{29}O_5N_4$	345.2119	345.2132	-3.9	345.2124, 310.1754, 286.1635, 264.1701, 186.1230, 175.1179, 158.0916, 112.0869, **70.0674**;	Azelayl arginine isomer	[4]
27	Alkaloids	19.525	$[M+H]^+$	$C_{11}H_{11}O_2N_2$	203.0807	203.0815	-4	203.0811, 188.0932, **160.0752**, 159.0667, 146.0592, 132.0797, 117.0575, 115.0544;	Unknown	-
28	Amino acids	19.724	$[M+H]^+$	$C_{15}H_{29}O_5N_4$	345.212	345.2132	-3.6	345.2126, 310.1753, 292.1656, 286.1645, 264.1704, 186.1233, 175.1189, 158.0922, 112.0876, **70.0681**;	Azelayl arginine isomer	[4]
29	_	20.304	$[M+H]^+$	$C_{15}H_{13}ON_2$	237.1014	237.1022	-3.5	237.1019, 235.0855, **222.0780**, 221.0701, 193.0753;	Unknown	-
30	_	20.764	$[M+H]^+$	$C_{17}H_{21}O_6N_4$	377.1441	377.1456	-3.9	377.1432, 359.1336, **243.0852**, 198.0649, 172.0859, 160.0758, 69.0356;	Unknown	-

31	Amino acids	21.118	$[M+H]^+$	$C_{15}H_{29}O_5N_4$	345.2119	345.2132	-3.9	345.2119, 278.1493, **250.1545**, 175.1186, 158.0922, 70.0683;	Azelayl arginine	[4]
32	Bufogenins	21.873	$[M+NH4]^+$	$C_{24}H_{34}O_5N$	416.2415	416.2431	-4	416.2413, **398.2307**, 380.2193, 370.2357, 363.1942, 345.1836, 317.1879, 237.0900, 131.0851;	Resibufagin stereoisomer	[6]
33	Alkaloids	22.008	$[M+H]^+$	$C_{14}H_{17}O_4N_2$	277.1173	277.1183	-3.5	277.1177, 259.1055, **160.0753**, 132.0807, 117.0583, 115.0547, 105.0710;	Unknown	-
34	Amino acids	23.756	$[M+H]^+$	$C_{16}H_{31}O_5N_4$	359.2277	359.2289	-3.3	359.2278, 342.2007, 324.1907, 250.1546, **186.1232**, 70.0678;	Sebacyl arginine isomer	[4]
35	Amino acids	25.159	$[M+H]^+$	$C_{16}H_{31}O_5N_4$	359.2275	359.2289	-3.9	359.2271, 342.2014, **278.1848**, 175.1180, 158.0920, 70.0679;	Sebacyl arginine isomer	[4]
36	_	28.206	$[M+H]^+$	$C_{25}H_{36}O_5N$	430.257	430.2588	-4.2	430.2572, 412.2468, **366.2053**, 363.1942, 189.1269, 171.1165;	Unknown	-
37	Amino acids	29.003	$[M+H]^+$	$C_{16}H_{31}O_5N_4$	359.2278	359.2289	-3.1	359.2281, 278.1499, **250.1550,** 175.1192, 158.0927, 139.0759, 70.0685;	Sebacyl arginine	[1,4]

38	_	30.614	$[M+H]^+$	$C_{23}H_{36}O_3N$	374.2677	374.269	-3.4	374.2688, **356.2577**, 338.2472, 177.1148;	Unknown	-
39	Bufogenins	31.797	$[M+H]^+$	$C_{24}H_{31}O_6$	415.2098	415.2115	-4.1	415.2101, **397.2028**, 351.1958, 333.1800, 256.1049, 147.0796, 133.1013;	19-oxo-Desacetylcinobufagin	[6]
40	Bufogenins	32.309	$[M+H]^+$	$C_{24}H_{35}O_6$	419.2412	419.2428	-3.9	419.2420, **401.2306**, 383.2183, 365.2097, 347.1987, 337.2159;	Hellebrigenol	[1,6]
41	Bufogenins	33.064	$[M+H]^+$	$C_{24}H_{33}O_6$	417.2261	417.2272	-2.6	417.2261, 399.2322, 381.2047, 363.1945, 351.1930, 335.1991, 321.1822, 249.1631, **145.1004**;	Hellebrigenol-9,11-ene	[7]
42[b]	Amino acids	33.276	$[M+H]^+$	$C_{17}H_{33}O_5N_4$	373.2433	373.2445	-3.3	373.2419, 356.2161, 324.1912, 264.1690, 250.1533, **186.1228**, 175.1180, 158.0918, 70.0669;	Undecanedioyl arginine	[4]
43[b]	Bufotoxins	33.475	$[M+H]^+$	$C_{34}H_{49}O_{10}N_4$	673.3415	673.3443	-4.2	673.3420, 655.3225, 399.2167, 335.2018, **275.1340**, 175.1191;	3-(N-succinyl argininyl) arenobufagin	-
44	Bufogenins	34.119	$[M+H]^+$	$C_{24}H_{35}O_6$	419.2413	419.2428	-3.6	419.2417, **401.2303**, 383.2203, 347.2014, 147.1162;	Hellebrigenol isomer	[1,6]

45	Amino acids	34.247	$[M+H]^+$	$C_{17}H_{33}O_5N_4$	373.2433	373.2445	-3.3	373.2430, 356.2183, 310.1744, 278.1480, **172.1073**, 139.0749, 83.0858, 70.0672;	Undecanedioyl arginine isomer	[4]
46[b]	Bufotoxins	35.513	$[M+H]^+$	$C_{35}H_{51}O_{10}N_4$	687.3579	687.36	-3	687.3570, 669.3443, 335.1978, **289.1495**, 175.1194;	3-(N-glutaryl argininyl) arenobufagin	-
47[a]	Bufogenins	35.595	$[M+H]^+$	$C_{24}H_{33}O_6$	417.226	417.2272	-2.8	417.2250, **399.2154**, 371.2206, 335.1994, 317.1892, 147.0798;	Ψ-Bufarenogin	[1]
48	Bufogenins	35.87	$[M+H]^+$	$C_{26}H_{35}O_8$	475.2307	475.2326	-4.1	475.2328, **415.2114**, 397.1997, 379.1901, 361.1808, 333.1844, 271.0960, 259.0966, 225.0907, 175.0751;	5, 12β-dihydroxycinobufagin	[1,8]
49[b]	Bufotoxins	35.886	$[M+H]^+$	$C_{34}H_{49}O_{10}N_4$	673.3414	673.3443	-4.3	673.3394, 655.3172, 399.2144, **275.1339**, 175.1178;	3-(N-succinyl argininyl) hellebrigenin	-
50	Bufogenins	35.966	$[M+H]^+$	$C_{24}H_{31}O_7$	431.205	431.2064	-3.3	431.2051, 349.1792, 313.1574, 199.1470, 157.1013, **143.0847**;	Unknown	-
51	Bufotoxins	36.06	$[M+H]^+$	$C_{36}H_{53}O_{10}N_4$	701.3727	701.3756	-4.2	701.3715, 683.4118, 399.2153, 363.1948, **303.1639**, 250.1158, 175.1174, 158.0906;	3-(N-adipoyl argininyl) arenobufagin	[1,9]

52	Bufogenins	36.271	$[M+H]^+$	$C_{24}H_{35}O_6$	419.2415	419.2428	-3.1	419.2410, 401.2302, 383.2198, 365.2097, **347.1985**, 251.1786, 197.1314, 145.1010, 131.0852;	Hellebrigenol stereoisomer	[1,6]
53	Bufogenins	36.413	$[M+H]^+$	$C_{24}H_{31}O_6$	415.2103	415.2115	-2.9	415.2100, **397.1994**, 379.1895, 351.1990, 257.1523, 225.0900;	Bufotalinin isomer	[10]
54[a]	Bufogenins	37.134	$[M+H]^+$	$C_{24}H_{35}O_5$	403.247	403.2479	-2.2	403.2466, 385.2367, 367.2259, **349.2155**, 331.2051, 271.2048, 253.1944, 145.1008;	Gamabufotalin	[10-12]
55	Bufogenins	37.608	$[M+H]^+$	$C_{26}H_{35}O_8$	475.231	475.2326	-3.5	475.2297, **415.2097**, 397.1991, 379.1889, 361.1786, 333.1843, 175.0745;	Gamabufotalin-3-oxalate	[1,7]
56	Bufogenins	37.914	$[M+H]^+$	$C_{24}H_{33}O_6$	417.226	417.2272	-2.8	417.2266, **399.2161**, 381.2064, 371.2214, 335.2004, 317.1899, 307.2052, 255.1017;	Bufarenogin	[12]
57[b]	Bufotoxins	38.037	$[M+H]^+$	$C_{35}H_{51}O_{10}N_4$	687.3567	687.36	-4.8	687.3577, 669.3477, 303.1617, **289.1503**, 250.1178, 175.1193, 158.0922;	3-(N-glutaryl argininyl) hellebrigenin	-

58[b]	Bufotoxins	38.182	$[M+H]^+$	$C_{36}H_{53}O_{10}N_4$	701.3728	701.3756	-4	701.3721, 699.1835, 683.3567, 399.2148, 363.1957, **303.1655**, 286.1412, 268.1305, 250.1181, 175.1176;	3-(N-adipoyl argininyl) hellebrigenin or isomer	-
59	Bufotoxins	38.452	$[M+H]^+$	$C_{36}H_{55}O_9N_4$	687.3934	687.3964	-4.3	687.3928, 669.3826, **303.1644**, 250.1173, 226.1067, 175.1181;	3-(N-adipoyl argininyl) Gamabufotalin isomer	[1]
60	Bufogenins	38.496	$[M+H]^+$	$C_{24}H_{35}O_5$	403.247	403.2479	-2.2	403.2473, **385.2365**, 367.2251, 349.2144, 339.2316, 271.2040, 253.1949, 147.1171, 145.1012;	19-hydroxylbufalin	[6]
61	Bufotoxins	38.966	$[M+H]^+$	$C_{36}H_{55}O_9N_4$	687.3934	687.3964	-4.3	687.3936, **303.1653**, 250.1190, 175.1179;	3-(N-adipoyl argininyl) Gamabufotalin isomer	[1]
62	Bufogenins	40.284	$[M+H]^+$	$C_{24}H_{35}O_6$	419.2414	419.2428	-3.4	419.2413, 365.2077, 353.2081, 347.1975, **335.1986**, 251.1779, 161.1324, 145.1003;	Hellebrigenol stereoisomer	[1,6]
63[b]	Bufotoxins	40.345	$[M+H]^+$	$C_{38}H_{59}O_{10}N_4$	731.4193	731.4226	-4.5	731.4184, 713.4110, 695.3990, **331.1964**, 250.1540, 158.0899;	3-(N-suberoyl argininyl) hellebrigenol	-
64	Bufotoxins	40.836	$[M+H]^+$	$C_{36}H_{55}O_9N_4$	687.3938	687.3964	-3.7	687.3934, 669.3860, **303.1643,** 250.1167, 175.1176;	3-(N-adipoyl argininyl) gamabufotalin	[1,8]

65	Bufogenins	40.886	$[M+H]^+$	$C_{24}H_{35}O_6$	419.2415	419.2428	-3.1	419.2426, 401.2303, 383.2212, **365.2106**, 347.1990, 337.2096, 245.1885;	11α-hydroxytelocinobufagin	[8]
66	Bufotoxins	41.429	$[M+H]^+$	$C_{34}H_{49}O_8N_4$	641.3523	641.3545	-3.4	641.3531, **257.1240**, 240.0980, 198.0748;	Unknown	-
67	Bufotoxins	42.487	$[M+H]^+$	$C_{36}H_{53}O_{10}N_4$	701.378	701.3756	3.4	701.4079, 683.3770, **317.1810,** 264.1330, 175.1182, 158.0914;	3-(N-pimeloyl argininyl) gamabufotalin isomer	[8,11]
68	Bufotoxins	42.818	$[M+H]^+$	$C_{36}H_{55}O_9N_4$	687.3939	687.3964	-3.6	687.3939, 669.3807, 349.2151, **303.1647**, 250.1190;	3-(N-adipoyl argininyl) gamabufotalin isomer	[1,8]
69[b]	Bufotoxins	43.061	$[M+H]^+$	$C_{37}H_{55}O_{10}N_4$	715.3887	715.3913	-3.6	715.3893, 697.3703, 399.2141, 381.2065, **317.1799**, 282.1445, 264.1328, 236.1371, 175.1179, 158.0917;	3-(N-pimeloyl argininyl) arenobufagin	-
70	Bufotoxins	43.112	$[M+H]^+$	$C_{37}H_{57}O_9N_4$	701.4095	701.412	-3.6	701.4092, 683.3992, 665.3904, **317.1812,** 264.1337, 236.1385, 175.1181, 158.0917;	3-(N-pimeloyl argininyl) gamabufotalin	[8,11]
71[a]	Bufogenins	43.27	$[M+H]^+$	$C_{24}H_{33}O_6$	417.2262	417.2272	-2.3	417.2265, **399.2163**, 371.2212, 335.2000, 307.2055, 289.1935, 255.1013, 175.0747;	Arenobufagin	[7,11]

72[b]	Bufotoxins	44.285	$[M+H]^+$	$C_{36}H_{51}O_{11}N_4$	715.3523	715.3549	-3.6	715.3530, 697.3396, 381.2055, **275.1338**, 258.1082, 240.0971, 175.1171;	3-(N-succinyl argininyl) cinobufaginol or isomer	-
73	Bufogenins	44.559	$[M+H]^+$	$C_{26}H_{35}O_7$	459.2362	459.2377	-3.3	459.2369, 399.2166, 363.1923, **335.1968;**	Cinobufotalin isomer	[6]
74	Bufogenins	44.722	$[M+H]^+$	$C_{24}H_{33}O_6$	417.2259	417.2272	-3	417.2249, 381.2047, 363.1944, **335.1994**, 317.1886, 289.1942, 275.1779, 239.1782;	Hellebrigenin	[2,6]
75[b]	Bufotoxins	45.246	$[M+H]^+$	$C_{37}H_{55}O_{10}N_4$	715.3892	715.3913	-2.9	715.3881, 697.3766, **317.1811**, 264.1344, 175.1177, 145.0996;	3-(N-pimeloyl argininyl) hellebrigenin	-
76	Bufotoxins	45.254	$[M+H]^+$	$C_{36}H_{53}O_{10}N_4$	701.3742	701.3756	-2	701.3774, 683.3977, 623.3430, 349.2144, 317.1814, **275.1344**, 240.0981, 175.1186, 158.0921;	3-(N-succinyl argininyl) bufotalin	[11]
77	Bufotoxins	45.378	$[M+H]^+$	$C_{38}H_{57}O_{10}N_4$	729.4043	729.4069	-3.6	729.4051, 711.3942, 399.2146, **331.1970**, 278.1497, 250.1541, 175.1186;	3-(N-suberoyl argininyl) arenobufagin (Arenobufotoxin)	[1]
78	Bufogenins	45.382	$[M+H]^+$	$C_{24}H_{35}O_5$	403.2468	403.2479	-2.7	403.2460, 385.2356, 367.2256, **349.2152**, 337.2149, 253.1942, 145.1009;	12-β-hydroxylbufalin	[6]

79	Bufotoxins	45.778	$[M+H]^+$	$C_{36}H_{55}O_9N_4$	687.3944	687.3964	-2.8	687.3947, 669.3831, 651.3701, **303.1651**, 250.1172;	3-(N-adipoyl argininyl) telocinobufagin	[1,4]
80	Bufogenins	46.246	$[M+H]^+$	$C_{24}H_{33}O_6$	417.226	417.2272	-2.8	417.2257, 399.2141, 381.2051, **363.1942**, 345.1835, 339.1942, 335.1991, 317.1884, 255.1007, 157.1007;	Desacetylcinobufotalin	[1,13]
81	Bufotoxins	46.355	$[M+H]^+$	$C_{38}H_{57}O_{10}N_4$	729.4044	729.4069	-3.5	729.4033, 711.3926, 670.3909, 381.2038, 363.1931, **331.1961**, 278.1485, 250.1532, 175.1177;	Arenobufotoxin isomer	[1]
82	Bufogenins	46.689	$[M+H]^+$	$C_{24}H_{31}O_6$	415.2103	415.2115	-2.9	415.2087, **397.2013**, 379.1870, 361.1788, 301.1765, 231.1373, 151.0745;	Bufotalinin isomer	[10]
83	Bufotoxins	46.935	$[M+H]^+$	$C_{38}H_{57}O_{10}N_4$	729.4044	729.4069	-3.5	729.4033, 711.3909, 693.3837, 683.3936, 665.3856, 363.1930, 335.1997, **331.1953**, 278.1490, 250.1536, 175.1169;	Arenobufotoxin isomer	[1]
84	Bufotoxins	47.07	$[M+H]^+$	$C_{38}H_{59}O_9N_4$	715.425	715.4277	-3.7	715.4253, 697.4130, **331.1972**, 278.1491, 250.1551;	3-(N-suberoyl argininyl) gamabufotalin (Gamabufotalitoxin)	[2,12]

85	Bufotoxins	48.251	$[M+H]^+$	$C_{37}H_{55}O_{10}N_4$	715.3893	715.3913	-2.8	715.3911, **317.1805**, 264.1321, 236.1376, 175.1185;	3-(N-pimeloyl argininyl) arenobufagin isomer	-
86	Bufogenins	48.365	$[M+H]^+$	$C_{26}H_{37}O_7$	461.252	461.2534	-3	461.2522, 401.2362, 383.2203, **365.2099**, 347.2005, 319.2065, 265.1200, 211.1459, 145.1009;	Monohydroxylbufotalin	[1]
87	Bufotoxins	49.22	$[M+H]^+$	$C_{38}H_{59}O_9N_4$	715.425	715.4277	-3.7	715.4252, 697.4121, 679.4070, **331.1956**, 289.1498, 278.1491, 250.1541, 175.1176;	Gamabufotalitoxin isomer	[2,12]
88	Bufogenins	49.293	$[M+H]^+$	$C_{26}H_{35}O_7$	459.2364	459.2377	-2.9	459.2353, 381.2039, **363.1938**, 351.1936, 345.1829, 335.1986, 151.0384;	Cinobufaginol	[11,13]
89	Bufotoxins	49.398	$[M+H]^+$	$C_{38}H_{57}O_{10}N_4$	729.4044	729.4069	-3.5	729.4042, 711.3934, 399.2156, 381.2059, **331.1969**, 278.1488, 250.1543, 175.1184;	3-(N-suberoyl argininyl) hellebrigenin (Hellebritoxin)	[8]
90	Bufogenins	49.947	$[M+H]^+$	$C_{24}H_{31}O_6$	415.2105	415.2115	-2.4	415.2104, 397.1976, 387.2154, 323.1988, 215.1038, 161.0955, **133.1002**, 91.0555;	3-keto-arenobufagin	[13]

91	Bufotoxins	50.131	$[M+H]^+$	$C_{38}H_{55}O_{10}N_4$	727.3888	727.3913	-3.4	727.3887, 709.3843, 397.1998, **331.1976**, 278.1528, 250.1526, 175.1196;	3-(N-suberoyl argininyl) bufotalinin	[1,4]
92	Bufotoxins	50.228	$[M+H]^+$	$C_{40}H_{59}O_{11}N_4$	771.4151	771.4175	-3.1	771.4157, 345.1849, **331.1968**, 278.1476, 250.1537;	3-(N-suberoyl argininyl) cinobufaginol	[11]
93	Bufogenins	50.442	$[M+H]^+$	$C_{24}H_{33}O_5$	401.2312	401.2323	-2.6	**383.2208**, 365.2090, 347.1999, 337.2149;	Resibufaginol	[6]
94	Bufotoxins	50.799	$[M+H]^+$	$C_{37}H_{57}O_9N_4$	701.4094	701.412	-3.7	701.4100, 683.3987, 665.3911, **317.1813**, 264.1338, 175.1187;	3-(N-pimeloyl argininyl) telocinobufagin	[1,4]
95	Bufotoxins	51.69	$[M+H]^+$	$C_{38}H_{55}O_{10}N_4$	727.3886	727.3913	-3.7	727.3878, 709.3747, **331.1923**, 250.1539;	3-(N-suberoyl argininyl) bufotalinin isomer	[1,4]
96	Bufogenins	51.812	$[M+H]^+$	$C_{24}H_{31}O_6$	415.2106	415.2115	-2.2	415.2106, 397.2010, 379.1895, 361.1783, **351.1938**, 343.1688, 333.1838, 237.1635, 145.1006, 143.0852;	Bufotalinin	[6,10]
97	Bufotoxins	51.975	$[M+H]^+$	$C_{38}H_{59}O_9N_4$	715.4253	715.4277	-3.3	715.4257, 697.4149, 349.2157, **331.1976**, 278.1503, 250.1555, 175.1179;	Gamabufotalitoxin isomer	[2,12]
98	Bufotoxins	52.494	[M+H]+	C34H51O8N4	643.3679	643.3701	-3.5	643.3690, 625.3583, 351.2299, **275.1348**, 240.0977, 175.1184,	3-(N-succinyl-argininyl) bufalin	[2]

								140.0812, 100.0395, 95.0846;		
99	Bufogenins	52.7	$[M+H]^+$	$C_{24}H_{35}O_5$	403.2464	403.2479	-3.7	403.2479, 385.2373, **367.2254**, 349.2136, 215.1791;	Desacetylbufotalin	[1,6]
100	Bufogenins	52.767	$[M+H]^+$	$C_{24}H_{33}O_5$	401.2312	401.2323	-2.6	383.2183, **365.2098**, 355.2292, 347.1982, 337.2139, 319.2035, 251.1792;	12-β-hydroxylresibufogenin	[6]
101	Bufogenins	53.756	$[M+H]^+$	$C_{26}H_{33}O_8$	473.2157	473.217	-2.7	473.2164, 395.1845, 377.1736, 367.1905, **349.1798**, 331.1691, 321.1852, 235.1473, 217.1576;	19-oxo-cinobufotalin	[1]
102	Bufotoxins	54.056	$[M+H]^+$	$C_{40}H_{59}O_{11}N_4$	771.4142	771.4175	-4.3	771.4146, 753.4033, 423.2124, 363.1950, **278.1490**, 250.1551, 175.1188;	3-(N-suberoyl argininyl) cinobufaginol isomer	[11]
103	Bufogenins	54.484	$[M+H]^+$	$C_{24}H_{35}O_5$	403.2465	403.2479	-3.5	403.2469, 385.2359, 367.2257, **349.2152**;	1-β-hydroxylbufalin	[6]
104	Bufotoxins	54.509	$[M+H]^+$	$C_{38}H_{55}O_{10}N_4$	727.3888	727.3913	-3.4	727.3888, 343.1977, **331.1968**, 278.1485, 250.1550, 175.1198;	3-(N-suberoyl argininyl) bufotalinin isomer	[1,4]
105	Bufotoxins	55.215	$[M+H]^+$	$C_{38}H_{57}O_{10}N_4$	729.4041	729.4069	-3.9	729.4043, 711.3952, 399.2167, **331.1972**,	Arenobufotoxin isomer	[1]

								278.1494, 250.1546, 175.1187;		
106	Bufogenins	55.353	$[M+H]^+$	$C_{24}H_{33}O_5$	401.231	401.2323	-3.1	383.2206, 365.2100, 347.1999, **337.2156**, 319.2051;	19-oxo-bufalin	[2,6]
107	Bufogenins	56.632	$[M+H]^+$	$C_{24}H_{31}O_6$	415.2101	415.2115	-3.4	415.2111, **397.2008**, 379.1893, 361.1792, 351.1947, 343.1685, 333.1850, 175.0754;	Argentinogenin	[1]
108	Bufotoxins	56.672	$[M+H]^+$	$C_{39}H_{56}O_9N_3$	710.3982	710.4011	-4.1	710.3998, 692.3865, 674.3792, **326.1702**, 280.1642, 170.0914;	19-oxocinobufotalin-3-suberate methylhistidine	[1]
109	Bufotoxins	56.988	$[M+H]^+$	$C_{38}H_{59}O_9N_4$	715.425	715.4277	-3.7	715.4233, 697.4133, 679.4024, **331.1963**, 278.1485, 250.1535;	3-(N-suberoyl argininyl) telocinobufagin (Telocinobufatoxin)	[2,4]
110[a]	Bufogenins	59.509	$[M+H]^+$	$C_{24}H_{35}O_5$	403.2467	403.2479	-3	403.2465, 385.2364, 367.2259, **349.2155**, 331.2050, 253.1945, 215.1789, 159.1163, 145.1009;	Telocinobufagin	[7,8,10]
111	Bufogenins	59.539	$[M+H]^+$	$C_{26}H_{35}O_7$	459.236	459.2377	-3.8	417.2238, 399.2155, **381.2042**, 363.1943, 345.1833;	12β-hydroxylcinobufagin	[6]

112	Bufotoxins	59.659	$[M+H]^+$	$C_{38}H_{55}O_{10}N_4$	727.3885	727.3913	-3.8	727.3868, 709.3639, **331.1969**, 278.1478, 250.1523, 175.1173;	3-(N-suberoyl argininyl) bufotalinin isomer	[1,4]
113	Bufotoxins	60.728	$[M+H]^+$	$C_{40}H_{57}O_{12}N_4$	785.3942	785.3968	-3.2	785.3931, 767.3826, 377.1756, 359.2299, 349.1775, **331.1973**, 278.1514, 250.1537, 175.1189;	3-(N-suberoyl argininyl) 19-oxo-cinobufotalin	[1,11]
114	Bufotoxins	61.279	$[M+H]^+$	$C_{34}H_{49}O_8N_4$	641.352	641.3545	-3.9	641.3517, 623.3416, 367.2257, **275.1345**, 258.1079, 175.1188, 140.0821;	3-(N-succinyl argininyl) resibufogenin	[8]
115	Bufotoxins	61.334	$[M+H]^+$	$C_{38}H_{57}O_9N_4$	713.4092	713.412	-3.9	713.4088, **695.3972**, 331.1963, 278.1489, 250.1539, 175.1182;	Marinobufotoxin isomer	[4]
116	Bufotoxins	62.19	$[M+H]^+$	$C_{40}H_{59}O_{11}N_4$	771.4151	771.4175	-3.1	771.4137, 753.4024, 711.3872, 693.3837, 381.2043, **331.1960**, 278.1480, 250.1528, 175.1181;	3-(N-suberoyl argininyl) cinobufotalin	[1,11]
117	Bufogenins	62.362	$[M+H]^+$	$C_{24}H_{33}O_5$	401.2311	401.2323	-2.9	401.2313, 383.2206, **365.2101**, 347.1997, 323.2001, 319.2042, 257.1163, 251.1778;	Marinobufagin	[7,8,13]

118	Bufotoxins	62.511	$[M+H]^+$	$C_{38}H_{57}O_9N_4$	713.4092	713.412	-3.9	713.4090, 695.3970, 677.3854, **331.1968**, 278.1486, 250.1536;	3-(N-suberoyl argininyl) marinobufagin (Marinobufotoxin)	[4]
119	Bufotoxins	62.549	$[M+H]^+$	$C_{36}H_{51}O_{10}N_4$	699.3575	699.36	-3.5	699.3574, 681.3472, 639.3372, 425.2316, **275.1343**, 240.0972, 175.1186;	3-(N-succinyl argininyl) cinobufagin	[2,12]
120	Bufotoxins	63.364	$[M+H]^+$	$C_{36}H_{53}O_9N_4$	685.3786	685.3807	-2.6	685.3790, 667.3681, **319.1603**, 284.1209, 242.1026, 196.0947, 175.1181;	3-(N-adipoyl argininyl) marinobufagin	[4]
121[a]	Bufogenins	63.59	$[M+H]^+$	$C_{26}H_{37}O_6$	445.2569	445.2585	-3.5	445.2566, 409.2361, 367.2253, **349.2146**, 241.1216, 161.1318;	Bufotalin	[7,11,14]
122	Bufotoxins	63.603	$[M+H]^+$	$C_{36}H_{55}O_8N_4$	671.3993	671.4014	-3.2	671.3975, 653.3872, 351.2299, **303.1650**, 268.1282, 250.1176, 175.1181, 158.0917;	3-(N-adipoyl argininyl) bufalin	[1]
123	Bufogenins	63.669	$[M+H]^+$	$C_{24}H_{31}O_3$	367.2256	367.2268	-3.2	367.2255, **349.2154**, 331.2040, 267.1368, 253.1216, 241.1209, 131.0856;	Scillaridin A stereoisomer	[4]
124	Bufotoxins	65.411	$[M+H]^+$	$C_{38}H_{55}O_{10}N_4$	727.3891	727.3913	-3	727.3895, 709.3799, 681.3833, 397.1984, **331.1971**, 278.1505, 250.1538, 175.1189;	3-(N-suberoyl argininyl) bufotalinin isomer	[1,4]

125	Bufogenins	66.003	$[M+H]^+$	$C_{24}H_{31}O_5$	399.2155	399.2166	-2.8	399.2153, 381.2046, 363.1945, 353.2098, 345.1841, 335.1991, **257.1890**, 239.1784;	Resibufagin	[7,11]
126	Bufotoxins	66.174	$[M+H]^+$	$C_{35}H_{51}O_8N_4$	655.3678	655.3701	-3.6	655.3691, 637.3552, 504.3245, **289.1499**, 236.1021, 175.1180, 140.0806;	3-(N-glutaryl argininyl) resibufogenin	[12]
127	Bufotoxins	66.74	$[M+H]^+$	$C_{38}H_{59}O_9N_4$	715.4256	715.4277	-2.9	715.4247, **697.4153**, 347.1913, 312.1558, 276.1338, 269.1497, 251.1378, 175.1181, 158.0911;	Gamabufotalitoxin isomer	[2,12]
128	Bufogenins	66.809	$[M+H]^+$	$C_{26}H_{33}O_7$	457.2209	457.2221	-2.6	457.2208, 397.2007, 379.1886, 351.1939, 333.1830, 309.1467, 257.1159, 255.1730, 201.1631, **151.0382**;	19-oxo-cinobufagin	[6,10,12]
129	Bufotoxins	67.505	$[M+H]^+$	$C_{38}H_{55}O_9N_4$	711.3936	711.3964	-3.9	711.3937, 693.3789, **331.1945**, 278.1484, 250.1530, 175.1193, 158.0907;	3-(N-suberoyl argininyl) resibufagin	[1]
130	Bufotoxins	67.605	$[M+H]^+$	$C_{38}H_{57}O_9N_4$	713.4093	713.412	-3.8	713.4094, 695.3962, **331.1961**, 278.1488, 250.1536, 175.1176, 158.0919;	Marinobufotoxin isomer	[4]

131	Bufotoxins	67.774	$[M+H]^+$	$C_{40}H_{61}O_{10}N_4$	757.436	757.4382	-2.9	757.4368, 739.4272, 679.4061, 349.2158, **331.1979**, 278.1499, 250.1549, 175.1188, 158.0926;	3-(N-suberoyl argininyl) bufotalin (Bufotoxin)	[11]
132[a]	Bufogenins	67.903	$[M+H]^+$	$C_{26}H_{35}O_7$	459.2361	459.2377	-3.6	459.236, 417.2268, 381.2048, **363.1944**, 345.1848, 255.1012, 201.1630;	Cinobufotalin	[8,12-14]
133	Bufogenins	68.059	$[M+H]^+$	$C_{24}H_{33}O_5$	401.2308	401.2323	-3.6	401.2309, 383.2203, **365.2104**, 347.1995, 339.1948, 337.2159, 319.2048, 269.1894, 253.1943;	Desacetylcinobufagin	[6,7]
134	Bufotoxins	68.436	$[M+H]^+$	$C_{37}H_{57}O_8N_4$	685.4142	685.4171	-4.2	685.4142, 667.4042, 649.3932, 351.2309, **317.1811**, 264.1331, 175.1182;	3-(N-pimeloyl argininyl) bufalin	[4]
135	Bufotoxins	69.211	$[M+H]^+$	$C_{36}H_{53}O_8N_4$	669.3833	669.3858	-3.7	669.3818, 651.3707, **303.1647**, 268.1270, 250.1182, 222.1214, 175.1184, 70.0660;	3-(N-adipoyl argininyl) scillarenin	[15]
136	Bufotoxins	69.35	$[M+H]^+$	$C_{38}H_{55}O_{10}N_4$	727.389	727.3913	-3.1	727.3891, 709.3771, 667.3684, 649.3626, **303.1653,** 268.1285, 250.1180, 175.1185;	3-(N-adipoyl argininyl) cinobufagin	[2,12]

137	Bufotoxins	71.347	$[M+H]^+$	$C_{38}H_{59}O_8N_4$	699.4302	699.4327	-3.9	699.4302, **681.4192**, 369.2411, 331.1963, 278.1488, 250.1540, 175.1180;	3-(N-suberoyl argininyl) bufalin (Bufalitoxin)	[1,4,11]
138	Bufotoxins	71.724	$[M+H]^+$	$C_{39}H_{57}O_{10}N_4$	741.4045	741.4069	-3.3	741.4048, 723.3956, 681.3830, 425.2308, 365.2104, **317.1813**, 264.1337, 175.1181;	3-(N-pimeloyl argininyl) cinobufagin	[1,11]
139	Bufotoxins	71.764	$[M+H]^+$	$C_{37}H_{55}O_8N_4$	683.399	683.4014	-3.6	683.3977, 665.3866, 532.3602, **317.1801**, 264.1330, 236.1382, 175.1177;	3-(N-pimeloyl argininyl) resibufogenin	[1,4]
140[a]	Bufogenins	73.524	$[M+H]^+$	$C_{24}H_{35}O_4$	387.2513	387.253	-3.1	387.2518, **369.2417**, 351.2310, 333.2207, 305.2252, 255.2102, 173.1314, 145.1006;	Bufalin	[10,13,14]
141	Hormone	73.595	$[M+H]^+$	$C_{24}H_{35}O_4$	387.2518	387.253	-3.1	387.2518, **369.2417**, 351.2310, 255.2101, 159.1158, 145.1006, 133.1005, 107.0858;	Algestone acetonide	[3]
142	Bufogenins	73.667	$[M+H]^+$	$C_{24}H_{35}O_4$	387.2517	387.253	-3.3	387.2513, **369.2414**, 351.2305, 333.2205, 255.2096;	3-epi-Bufalin	[13]
143	Bufogenins	73.694	$[M+H]^+$	$C_{27}H_{37}O_7$	473.2514	473.2534	-4.2	473.2507, 417.2250, 399.2156, 381.2053, **363.1960**, 345.1852;	Methylatedhydroxylcinobufagin	[1]

144	Bufotoxins	73.865	$[M+H]^+$	$C_{40}H_{59}O_{10}N_4$	755.42	755.4226	-3.4	755.4184, 737.4093, 695.3995, 677.3883, 365.2100, 347.1994, **331.1968**, 278.1488, 250.1542, 175.1183;	3-(N-suberoyl argininyl) cinobufagin (Cinobufotoxin)	[1,2]
145	Bufotoxins	74.034	$[M+H]^+$	$C_{38}H_{57}O_8N_4$	697.4146	697.4171	-3.6	697.4155, 679.4055, 546.3778, **331.1979**, 278.1497, 250.1546, 175.1185;	3-(N-suberoyl argininyl) resibufogenin (Resibufotoxin)	[2,11,12]
146	Bufotoxins	74.41	$[M+H]^+$	$C_{39}H_{59}O_8N_4$	711.4299	711.4327	-4	711.4307, 693.4134, 560.4027, **345.2126**, 267.1704, 264.1705, 189.1352, 126.1023;	3-(N-azelayl argininyl) resibufogenin	[1,4]
147	Bufogenins	74.621	$[M+H]^+$	$C_{26}H_{35}O_6$	443.241	443.2428	-4.1	443.2410, 401.2316, 383.2206, **365.2073**, 347.1969, 251.1802;	Cinobufagin isomer	[13]
148	Bufogenins	74.875	$[M+H]^+$	$C_{24}H_{31}O_5$	399.2154	399.2166	-3	399.2158, **381.2042**, 363.1913, 353.2094, 335.1984, 307.2059, 289.1950, 239.1778, 213.1624, 133.1010;	Resibufagin isomer	[13]
149[b]	Bufotoxins	75.926	$[M+H]^+$	$C_{41}H_{61}O_{10}N_4$	769.435	769.4382	-4.2	769.4362, 383.2217, **345.2120**, 292.1639, 264.1699, 175.1185;	3-(N-azelayl argininyl) cinobufagin	-

150	Bufogenins	76.286	$[M+H]^+$	$C_{24}H_{31}O_3$	367.2258	367.2268	-2.6	367.2257, **349.2155**, 241.1220, 161.1337, 131.0856;	Scillaridin A stereoisomer	[4]
151	Bufogenins	77.329	$[M+H]^+$	$C_{24}H_{31}O_5$	399.2154	399.2166	-3	399.2156, **381.2039**, 363.1932, 353.2091, 317.1882, 213.1271, 197.1308, 175.0749;	Resibufagin stereoisomer	[13]
152	Bufogenins	77.772	$[M+H]^+$	$C_{24}H_{31}O_4$	383.2202	383.2217	-3.9	383.2203, **365.2106**, 347.1990, 271.1319, 257.1151, 215.1798;	Unknown	-
153[a]	Bufogenins	77.804	$[M+H]^+$	$C_{26}H_{35}O_6$	443.2409	443.2428	-4.3	443.2405, 401.2312,383.2209, 365.2093, 347.1993, 251.1780, **187.1471**;	Cinobufagin	[6,7,12,14]
154	Bufotoxins	78.079	$[M+H]^+$	$C_{39}H_{59}O_8N_4$	711.4302	711.4327	-3.6	711.4299, 693.4440, **345.2119**, 310.1763, 285.1802, 278.1482, 172.1070, 139.0748;	3-(N-azelayl argininyl) resibufogenin isomer	[1,4]
155[a]	Bufogenins	78.739	$[M+H]^+$	$C_{24}H_{33}O_4$	385.2362	385.2373	-2.9	385.2356, 367.2257, 349.2154 , 339.2306, 253.1945, **145.1007**;	Resibufogenin	[6,7,14]
156	Bufogenins	78.764	$[M+H]^+$	$C_{24}H_{31}O_3$	367.2258	367.2268	-2.6	367.2255, **349.2162**, 339.2346, 331.2028, 321.2185, 303.2104,	Scillaridin A stereoisomer	[4]

								255.2081, 175.1474, 157.1002, 143.0850;		
157	Bufogenins	79.07	$[M+H]^+$	$C_{26}H_{35}O_6$	443.2413	443.2428	-3.4	443.2413, 401.2321, **365.2096**, 347.1988, 215.1794;	3-epi-Cinobufagin	[13]

[a] Confirmed by reference standards

[b] Detected in Chansu for the first time

References

[1] W. Ren, L.Y. Han, M.Y. Luo, et al., Multi-component identification and target cell-based screening of potential bioactive compounds in toad venom by UPLC coupled with high-resolution LTQ-Orbitrap MS and high-sensitivity Qtrap MS, Anal. Bioanal. Chem. 410 (2018) 4419-4435. https://doi.org/10.1007/s00216-018-1097-4

[2] J. Zhou, Y. Gong, H.Y. Ma, et al., Effect of drying methods on the free and conjugated bufadienolide content in toad venom determined by ultra-performance liquid chromatography-triple quadrupole mass spectrometry coupled with a pattern recognition approach, J. Pharm. Biomed. Anal. 114 (2015) 482-487. http://dx.doi.org/10.1016/j.jpba.2015.05.032

[3] A.H.M. Zulfiker, M. Sohrabi, J. Qi, et al., Multi-constituent identification in Australian cane toad skin extracts using high-performance liquid chromatography high-resolution tandem mass spectrometry, J. Pharm. Biomed. Anal. 129 (2016) 260-272. http://dx.doi.org/10.1016/j.jpba.2016.06.031

[4] G.S. Hirschmann, C. Quispe, G.V. Arana, et al., Antiproliferative activity and chemical composition of the venom from the Amazonian toad Rhinella marina (Anura: Bufonidae), Toxicon. 121 (2016) 119-129. http://dx.doi.org/10.1016/j.toxicon.2016.09.004

[5] C. Rodríguez, L.R. Smith, R. Ibáñez, et al., Toxins and pharmacologically active compounds from species of the family Bufonidae (Amphibia, Anura), J. Ethnopharmacol. 198 (2017) 235-254. http://dx.doi.org/10.1016/j.jep.2016.12.021

[6] M. Ye, D.A. Guo, Analysis of bufadienolides in the Chinese drug ChanSu by high-performance liquid chromatography with atmospheric pressure chemical ionization tandem mass spectrometry, Rapid Commun. Mass Spectrom. 19 (2005) 1881–1892. https://doi.org/

10.1002/rcm.1989
[7] Y.M. Hu, Z.L. Yu, Z.J. Yang, et al., Comprehensive chemical analysis of Venenum Bufonis by using liquid chromatography/electrospray ionization tandemmass spectrometry, J. Pharm. Biomed. Anal. 56 (2011) 210-220. http://dx.doi.org/10.1016/j.jpba.2011.05.014
[8] Q. Meng, L.F. Yau, J.G. Lu, et al., Chemical profiling and cytotoxicity assay of bufadienolides in toad Venom and toad skin, J. Ethnopharmacol. 187 (2016) 74–82. http://dx.doi.org/10.1016/j.jep.2016.03.062
[9] G. Schmeda-Hirschmann, C.V. Gomez, A. Rojas de Arias, et al., The Paraguayan Rhinella toad venom: Implications in the traditional medicine and proliferation of breast cancer cells, J. Ethnopharmacol. 199 (2017) 106–118. http://dx.doi.org/10.1016/j.jep.2017.01.047
[10] M. Ye, H. Guo, H.Z. Guo, et al., Simultaneous determination of cytotoxicbufadienolides in the Chinese medicine ChanSu by high-performance liquid chromatography coupled with photodiode array and mass spectrometry detections,J.Chromatogr. B. 838 (2006) 86–95. http://dx.doi.org/10.1016/j.jchromb.2006.04.042
[11] Y.M. Wang, Z.Y. Li, J.J. Wang, et al., Bufadienolides and polyhydroxycholestane derivatives from *Bufo bufo gargarizans*, J. Asian Nat. Prod. Res. 2015 (17) 364–376. http://dx.doi.org/10.1080/10286020.2014.995174
[12] H.Y. Ma, H.X. Niu, Q. Cao, et al., Metabolomics method based on ultra high performance liquid chromatography with time-of-flight mass spectrometry to analyze toxins in fresh and dried toad venom, J. Sep. Sci. 39 (2016) 4681-4687. https://doi.org/10.1002/jssc.201600827
[13] Y. Zhang, H.L. Jin, X.L. Li, et al., Separation and characterization of bufadienolides in toad skin using two-dimensional normal-phase liquid chromatography × reversed-phase liquid chromatography coupled with mass spectrometry, J.Chromatogr. B. 1026 (2016) 67–74. http://dx.doi.org/10.1016/j.jchromb.2015.11.015
[14] H.M. Gao, M. Zehl, A. Leitner, et al., Comparison of toad venoms from different *Bufo species* by HPLC and LC-DAD-MS/MS, J. Ethnopharmacol. 131 (2010) 368–376. http://dx.doi.org/10.1016/j.jep.2010.07.017
[15] T. Tanase, A. Nagatsu, N. Murakami, et al., Studies on Cardiac Ingredients of Plants. XI. Synthesis of New Bufotoxin Homologues Utilizing Scillarenin (the Genuine Aglycone of Proscillaridin), and Their Biological Activities, Chem. Pharm. Bull. 42 (1994) 2256-2262. https://doi.org/10.1248/cpb.42.2256

Table S2. Similarity results of 20 batches of Chansu samples.

	AH1	AH2	AH3	AH4	HN1	HN2	HN3	HN4	JX1	JX2	JX3	JX4	SC1	SC2	SC3	SC4	ZJ1	ZJ2	ZJ3	ZJ4	R
AH1	1.000																				
AH2	0.992	1.000																			
AH3	0.974	0.960	1.000																		
AH4	0.950	0.932	0.982	1.000																	
HN1	0.954	0.938	0.992	0.982	1.000																
HN2	0.953	0.940	0.990	0.979	0.997	1.000															
HN3	0.953	0.938	0.988	0.979	0.996	0.996	1.000														
HN4	0.955	0.940	0.993	0.982	0.999	0.997	0.997	1.000													
JX1	0.940	0.932	0.973	0.953	0.964	0.970	0.964	0.966	1.000												
JX2	0.942	0.937	0.970	0.950	0.962	0.969	0.962	0.964	0.998	1.000											
JX3	0.915	0.907	0.967	0.956	0.967	0.973	0.965	0.968	0.994	0.990	1.000										
JX4	0.919	0.910	0.966	0.951	0.966	0.971	0.964	0.968	0.995	0.992	0.998	1.000									
SC1	0.643	0.625	0.739	0.699	0.776	0.779	0.781	0.770	0.725	0.723	0.744	0.750	1.000								
SC2	0.722	0.717	0.818	0.789	0.839	0.844	0.845	0.836	0.784	0.782	0.800	0.795	0.940	1.000							
SC3	0.645	0.632	0.747	0.712	0.769	0.771	0.775	0.766	0.693	0.690	0.709	0.703	0.930	0.985	1.000						
SC4	0.652	0.639	0.753	0.718	0.775	0.777	0.781	0.772	0.700	0.698	0.716	0.711	0.933	0.987	1.000	1.000					
ZJ1	0.909	0.905	0.945	0.917	0.946	0.955	0.949	0.948	0.982	0.985	0.975	0.980	0.784	0.816	0.728	0.736	1.000				
ZJ2	0.906	0.904	0.941	0.914	0.942	0.952	0.946	0.944	0.980	0.984	0.972	0.978	0.783	0.815	0.725	0.734	1.000	1.000			
ZJ3	0.923	0.924	0.942	0.908	0.934	0.942	0.934	0.935	0.979	0.984	0.964	0.968	0.722	0.767	0.674	0.683	0.992	0.991	1.000		
ZJ4	0.922	0.924	0.939	0.906	0.931	0.940	0.932	0.932	0.977	0.983	0.961	0.966	0.722	0.769	0.676	0.684	0.991	0.991	1.000	1.000	
R	0.938	0.929	0.982	0.959	0.985	0.988	0.986	0.985	0.974	0.974	0.973	0.973	0.833	0.891	0.823	0.829	0.974	0.972	0.958	0.957	1.000
